# Perioperative Management of Patients on Buprenorphine and Methadone: A Narrative Review

**DOI:** 10.4274/balkanmedj.galenos.2020.2020.5.2

**Published:** 2020-08-11

**Authors:** Yasmin Sritapan, Sean Clifford, Alexander Bautista

**Affiliations:** 1 Department of Anesthesiology and Perioperative Medicine, University of Louisville, Louisville, Kentucky, USA

**Keywords:** Buprenorphine, chronic pain, methadone, opioid use disorder, perioperative management, surgery

## Abstract

The opioid epidemic has emerged as a major health and social problem over the last few decades. An increasing number of patients with opioid use disorder are presenting for perioperative management. These patients are either on buprenorphine or methadone for the maintenance and treatment of opioid addiction or chronic pain. In the settings of acute pain, the optimal management of patients with opioid use disorder is challenging, and recovery can be jeopardized secondary to the unique pharmacology of these agents. The purpose of this narrative review is to summarize the existing studies on the perioperative management of patients who are using buprenorphine and methadone and provide guidance for the management of patients with opioid use disorder during the perioperative period.

In 2016, an estimated 50 million adults in the United States struggled with chronic pain. (1) The chronic pain dilemma has led to a rapid increase in the use of opioid analgesics, which subsequently resulted in a greater number of overdose deaths due to opioid misuse. In 2018, according to the Department of Health and Human Services, two million people in the United States had opioid use disorder (OUD) and an estimated 130 people died every day due to opioid overdose (2). Correspondingly, different states have promulgated regulations on safe prescription by limiting the amount of opioid prescription and initiating online prescription-monitoring programs to address the misuse of opioid analgesics (3).

Medications such as long-acting synthetic opioid methadone and partial opioid agonist buprenorphine have been found to be the effective treatments for OUDs. These medications aim to decrease the illicit opioid use and relapse and increase treatment engagement (4,5). It is paramount that these medications be continued at all times and not stopped prematurely (4). Therefore, it is essential that anesthesia providers know how to care for these potential patients in the perioperative period. Although there have been several institutionalized guidelines and pain management strategies to help these patients, there is still a lack of standardization and paucity of data to guide providers in addressing perioperative care for patients. The purpose of this narrative review is to discuss the pharmacologic profiles of buprenorphine and methadone in relation to OUDs and maintenance, highlight the existing literature on the perioperative management of patients with OUDs, and summarize recommendations for the optimal care of this subset of patients in hopes to help the guide providers in developing anesthetic regimen to optimize patient care.

## PHARMACOLOGY of BUPRENORHINE

Buprenorphine is a derivative of thebaine, which is a mixed opioid receptor modulator. It is highly lipophilic and has a large distribution volume (6). Its activity occurs at multiple receptor types. It is a partial agonist at the mu opioid receptor, which has an analgesic effect. As compared to other opioids, buprenorphine has a strong binding affinity to the mu opioid receptor with a slow dissociation from the receptor (7). Buprenorphine is also an antagonist at the kappa opioid receptor, which has been linked in the treatment of depression (7). Some patients who have been resistant to the standard treatment modalities for depression have responded positively to buprenorphine therapy. Buprenorphine’s ability to limit the psychomimetic, euphoric, and induced hyperalgesia effects as compared to other opioids has been attributed to its kappa receptor antagonism (8). Additionally, buprenorphine is an agonist at the delta receptor, and its effects are not clearly understood (7). Buprenorphine has low receptor-stimulating activity, despite having a high receptor affinity and binding capacity leading to less euphoria, respiratory depression, and sedation even in higher doses (9). At a minimum, 40%-50% of receptor occupancy is needed to abate withdrawal symptoms in opioid-dependent patients, thus causing concern as the traditional dosing of opioids may not be effective in providing analgesia in the perioperative period (10).

Buprenorphine’s half-life is widely variable and depends on the route of administration. It is poorly absorbed by the gastrointestinal system; hence, it is administered via sublingual, dermal, and parenteral routes. Its half-life is three hours when it is administered via intravenous (IV) route and can be 24-60 hours when it is administered sublingually (11). It is metabolized by the liver and the majority of it is excreted through bile (6). Buprenorphine has a quick onset of 30-60 minutes when it is administered sublingually and 5-15 minutes with IV administration (6). The usual dose of buprenorphine ranges from 2 to 32 milligram per day (mg/d) and the maximum effect occurs the dose between 16 and 32 mg/d (12). It provides effective analgesia at low to moderate doses and is 30 times more potent than morphine. An IV dose of 0.3 mg of buprenorphine is equivalent to approximately 10 mg of morphine (13).

## PHARMACOLOGY of METHADONE

Methadone is a long-acting synthetic opioid that is a full mu receptor agonist. It has been used both as an analgesic in severe pain and in the treatment of OUDs. When used for the treatment of OUD, the oral stock solution is often mixed with orange-colored solution or cherry syrup to prevent the aberrant use through the parenteral route. It is rapidly absorbed following oral administration, and its effects start between 15 and 45 minutes with a maximum plasma concentration of 2.5-4 hours after administration. Methadone is widely distributed in the body and it is highly bound to protein leading to cumulative effect and slow elimination (14). It has a long but variable half-life with mean estimates varying from 15 to 55 hours (15). Methadone undergoes biotransformation rather than conjugation in the liver, and its metabolites are cleared via the fecal route. (16) The accumulation of methadone in the system may lead to sedation and respiratory depression, especially upon the initiation of therapy (17).

An effective daily dose of methadone for OUDs varies between 60 and 120 mg/d (17). The purpose of high doses is to suppress the withdrawal symptoms and eliminate cravings with minimal side effects. Patients on a higher dose may develop a prolonged QT interval, which could lead to the development of torsade de pointes, especially if co-administered with drugs that inherently prolong QT (18). It is important to note that a daily methadone dose for the treatment of OUD is inadequate in providing acute pain relief, and additional medications and strategies are often required to mitigate the pain (19).

## SUMMARY of EVIDENCE

Current studies related to the use of buprenorphine and methadone for patients with OUDs presenting for surgery are mostly retrospective reviews, case reports, case series, and expert opinions. Table 1 provides a summary of these published studies.

### Summary of evidence for buprenorphine

Buprenorphine has several advantages over methadone for the treatment of OUDs. It was shown to have less potential for abuse, provides greater flexibility in prescribing, and can be managed as an office-based treatment option (20). Therefore, there is an increased utilization of buprenorphine for OUD treatment.

There are several strategies proposed to treat severe pain in patients on buprenorphine maintenance treatment. When planning to continue buprenorphine during the perioperative period (especially in emergent and major surgeries), it is advised to use the different types of oral opioids and/or IV opioid analgesics with the use of patient-controlled analgesia (PCA) titrated for adequate pain control (21,22). A study showed that patients on buprenorphine may often require higher doses of opioids to displace the avidly bound buprenorphine from the mu opioid receptors and pain control can be adequately achieved (23). Alternatively, buprenorphine can be transitioned to full opioid agonists three to five days before elective procedures to avoid the challenges of poorly controlled pain (24).

A multimodal pain management approach with the use of dexmedetomidine, remifentanil, and ketamine perioperatively has shown success in providing adequate pain control among patients taking buprenorphine for OUD (25,26). The use of regional anesthesia, though reasonable, was inconsistent in terms of decreasing the amount of opioid used in the perioperative period in one case series (25). In patients who are on a low dose of buprenorphine, that is 2-8 mg per day, buprenorphine can be continued as an analgesic by increasing the frequency of the dose in every 6-8 hours (21). In patients who will not be able to tolerate sublingual buprenorphine, buprenorphine can be discontinued 72 hours before surgery and replaced with full mu opioid agonist for the titration of analgesia (21). If the risk of relapse is high, then buprenorphine can be replaced with methadone by using a modified dosing ratio of 1:5, respectively, and titrated to achieve adequate pain relief (21).

### Summary of evidence for methadone

Researchers have proposed algorithms for managing patients on methadone presenting for elective surgery. If the patient can tolerate oral medication, then it is advised to continue oral methadone on the morning of surgery and throughout the perioperative period (16,21,27). If the patient is unable to do so, then methadone can be dosed parenterally at a dose half to two-thirds of the maintenance dose divided into two to four equal doses a day (21). In situations when methadone is not available, the conversion of methadone to any opioid can be performed; however, the conversion calculations may not be bidirectional due to the long half-life of methadone (21). A multimodal approach (16,27) including the perioperative infusion of ketamine (28,29,30), clonidine (31), and the use of regional anesthesia (32) should be considered. In a retrospective cohort study performed by Macintyre et al. (26), methadone opioid substitution therapy was continued and it showed the efficacy and safety of PCA opioids for the management of postoperative pain (26). Nevertheless, caution is strongly advised while continuing methadone in specific surgeries such as bariatric as the serum levels of methadone can increase post-operatively (33).

## DISCUSSION

The perioperative management of patients with OUD is complex. These patients may have underlying medical, psychiatric, surgical conditions, and psychosocial problems in conjunction with their medication treatment. The management of their pain in the acute setting following surgery can be challenging and, often times, their OUD is not addressed. This may result with the worsening of their medical condition, readmission rates, overdose, and relapse. These patients require specific considerations and planning to prevent poor outcomes.

### Preoperative considerations

The stigma associated with OUDs may foster a negative judgment of the patient. These patients can be perceived as drug-seekers if their pain is poorly controlled. In this regard, patients worry about receiving inappropriate care in terms of pain control with the possibility of relapse and withdrawal symptoms.

It is recommended that these patients be evaluated preoperatively to obtain a full pain history, physical examination, and assessment for other psychiatric and medical comorbidities (34). It is imperative to take a detailed medication history that includes dose, frequency of ingestion, time of last dose, and assessment of the level of treatment stability (16). A urine toxicology screening provides useful information regarding medication compliance as well as the use of illicit drugs or medications beyond the agreement with their prescription provider. Opioid-monitoring databases should be accessed to review for controlled substance prescription. It is also prudent to reach out to the patient’s prescription provider to discuss the pain management strategy in the acute perioperative setting as well as arrange follow-up(s) for the continuation of maintenance therapy. The consultation of an in-house addiction specialist may be considered to provide support before and after the surgery. During the preoperative period, it is important to establish goals of care with the patient, which addresses realistic goals for pain management, the challenges of acute pain management as well as the concern and risk of relapse following the reinstitution of opioid therapy. It is important to educate patients and their family regarding the risk of relapse and appropriate management of anxiety, craving, and pain.

### Intraoperative considerations

It is important to understand that patients receiving maintenance therapy with buprenorphine and methadone do not receive adequate analgesia from these opioids in the setting of acute pain. The analgesic and addiction profile treatment differ as well as the associated neuroplastic changes associated with long-term opioid exposure, that is, tolerance and hyperalgesia may effectively diminish their analgesic properties (35). For example, in patients receiving methadone as maintenance therapy, the analgesic effect of morphine did not last as long as expected due to cross-tolerance (36). Patients also have significantly higher opioid requirements and a prolonged length of stay (23,37). This was echoed with a patient on buprenorphine therapy, who had poor pain control in the perioperative period in two different gynecological surgeries where buprenorphine was continued in the first and transitioned to full opioid agonist in the second (38). It may be beneficial to use regional anesthesia when possible and multimodal analgesic combinations to target pain pathways at different sites to provide superior pain relief and decreased opioid consumption.

### Postoperative considerations

The patient’s baseline addiction treatment needs to be addressed during the treatment of acute pain while taking maintenance buprenorphine and methadone therapy. It is imperative to have a plan for aggressive pain management (39). The undertreatment of pain predisposes patients to decreased opioid responsiveness, which can lead to difficulty in controlling pain (40). Analgesics including nonsteroidal anti-inflammatory drugs, acetaminophen, and adjuvant analgesics that can enhance opioid effects such as gabapentinoids and ketamine may be used together in a multimodal approach to improve the pain control (41,42,43).

If the decision is made to continue the use of methadone and buprenorphine, then it is important to continue the patient’s usual dose to avoid the worsening of pain symptoms due to increased pain sensitivity associated with opioid withdrawal (44). Adequate pain control would require the administration of a higher dose of opioid analgesic that should be continuous or scheduled, instead of prescribing it on a needed basis (39). The use of PCA was found to be helpful via increased control over analgesia and minimization of patient anxiety about pain management (45,46).

Regarding methadone, if the patient is unable to receive oral medications, then methadone dose can be administered via IV, intramuscular (IM) or subcutaneous (SC) routes. The dosing methadone via IV, IM and SC routes should be administered as half to two-thirds of the maintenance dose divided into two to four equal doses (47).

The high affinity of buprenorphine to the mu receptor makes pain treatment more complicated than methadone. The most effective approach comes with the clinical experience of the provider with buprenorphine. The upward titration of a short-acting full opioid agonist to effect is recommended if the decision is to continue buprenorphine in the perioperative period. The buprenorphine dose can also be increased in patients who are not on a maximal dose to 24-32 mg daily (20). Strategies that involve dividing the daily doses of buprenorphine to every six to eight hours have been discussed. A 0.4-mg dose of buprenorphine administered sublingually every eight hours may be adequate in patients who are opioid naïve (13). If the decision is made to discontinue buprenorphine therapy, then the patient should be treated with full opioid analgesics and withdrawal should be avoided (6). These patients should follow-up with the OUD provider in the postoperative period to resume maintenance therapy by using an induction protocol after the resolution of acute pain. Another strategy is to convert buprenorphine to methadone to prevent acute withdrawal (39).

### Summary of recommendations

**Figure 1:**
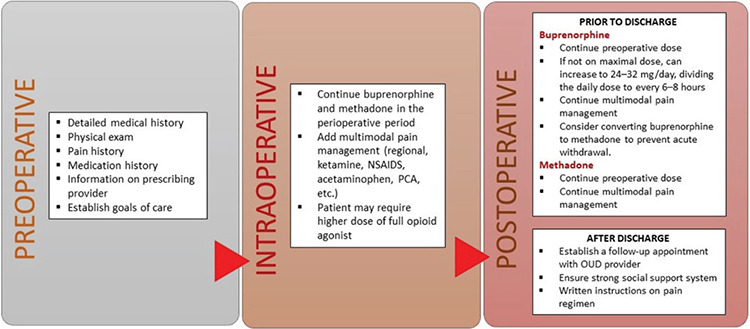
Outline of recommendation for the management of patients presenting for surgery with opioid use disorder.

Figure 1 outlines the authors’ recommendations in the management of patients with OUD. Patients with OUD should be seen in the preoperative clinic before the elective surgery to obtain: medical and pain history, medication dosage, and to better understand the patient’s support system outside of the hospital setting. In the setting of urgent or emergent surgeries, the same information and care discussion should be addressed before surgery if time permits. The ultimate goal is to achieve adequate pain management in patients with OUD receiving buprenorphine and methadone. We recommend continuing patients’ buprenorphine and methadone regimen in the perioperative setting with the understanding that patient may require higher amounts of opioid agonist. Our recommendation differs from other narrative reviews, which provide the option of discontinuing buprenorphine or methadone in the perioperative setting (43). Furthermore, multimodal analgesic strategies that incorporate the use of nonsteroidal anti-inflammatory drugs, acetaminophen, gabapentinoids, ketamine, alpha 2 agonist, and regional anesthesia should be utilized to achieve adequate pain management. Postoperative plan should be prepared before discharge, which incorporates written pain management regimen after discharge and follow-up with their outpatient provider of buprenorphine and methadone.

In conclusion, the increasing utilization of buprenorphine and methadone as a maintenance therapy for OUDs continues to grow to fight against the opioid epidemic. As a result, these patients face challenges in acute pain management during the perioperative period. These complex patients have altered the neural responses of tolerance and hyperalgesia that may alter and worsen the pain experience. Management with a traditional dosing of mu receptor opioid analgesics may be inadequate during the perioperative period. Moreover, patients may require higher doses of opioids with an inherent risk of precipitating patient cravings and subsequent relapse. Therefore, multimodal strategies should be employed as a part of the aggressive pain management to mitigate anxiety and allow for the successful treatment of pain. The author’s recommendation to continue buprenorphine and methadone was based on the strength of evidence and personal experience. However, providers should be cognizant of the fact that pain is subjective and treatment needs to be individualized.

## Figures and Tables

**Table 1:**
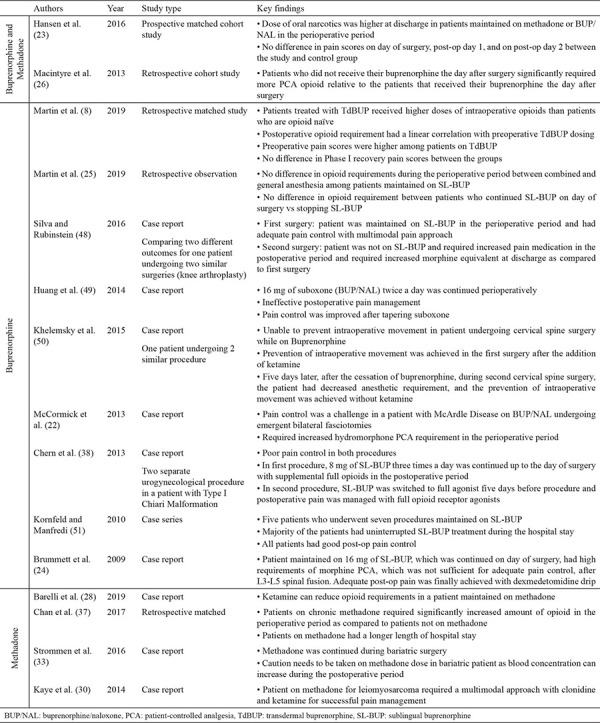
Summary of current studies published relating to the use of buprenorphine and methadone in patients with opioid use disorder presenting for surgery
